# Incidental Appendiceal Carcinoids: Is Surgery Affecting Their Incidence?

**DOI:** 10.4021/wjon400w

**Published:** 2012-10-28

**Authors:** Savio G. Barreto, Leong Tiong, Edward Travers, Randall S. Williams

**Affiliations:** Tudor Thomas; a Department of Surgery, Modbury Hospital, South Australia, Australia

**Keywords:** Tumors, Female, Child

## Abstract

**Background:**

There is lack of consensus on the incidence of appendiceal carcinoids in recent times. The influence of number of appendicectomies performed has been postulated to play a role in this. To determine the incidence and clinico-pathological profile of appendiceal carcinoids in a cohort of patients undergoing emergency appendicectomies for clinically suspected acute appendicitis, and examine the influence of the trend (if any) of the number of appendicectomies performed on the overall incidence of appendiceal carcinoids.

**Methods:**

A retrospective analysis of patients diagnosed with appendiceal carcinoids following presentation with acute appendicitis to the Modbury hospital, South Australia from March 2007 to April 2011. The patient cohort was divided into Group 1 (March 2007 - March 2009) and group 2 (April 2009 - April 2011) to study the influence of time trends on incidence of appendiceal carcinoids. Statistical analyses were performed using the Statistical Product and Service Solutions, SPSS 14.0 for Windows.

**Results:**

Of 506 patients who underwent emergency appendicectomy for acute appendicitis, 8 patients (1.6%) were found to have appendiceal carcinoids. The median age was 23 years with 7 patients being female. There was no difference in the incidence of appendiceal carcinoids over the two time periods (P < 0.12).

**Conclusions:**

Appendiceal carcinoids were found in 1.6% of patients undergoing emergency appendicectomy for acute appendicitis. These tumors are found more commonly in young females with a predilection for the tip of the appendix. The perceived increased incidence appendiceal carcinoids appear to be unrelated to the increase in the number of appendicectomies being performed.

## Introduction

Carcinoids are the most common tumors of the appendix [1]. However, their overall incidence amongst patients undergoing appendicectomies has been reported to be only between 0.3 and 0.9% [2]. Although long considered benign tumors with an indolent course, the malignant potential of carcinoid tumors is increasingly appreciated [3].

Recent literature on the incidence and prevalence of appendiceal carcinoids is contradictory with some studies reporting an increased incidence [4, 5], while other studies indicating a decrease in the overall prevalence [3] of these tumors. These trends were postulated to be linked to the number of appendicectomies performed [3, 4].

The aim of the current study was: 1) To determine the incidence and clinico-pathological profile of appendiceal carcinoids in a cohort of patients undergoing emergency appendicectomies for clinically suspected acute appendicitis; 2) To examine the influence of the trend (if any) of the number of appendicectomies performed on the overall incidence of appendiceal carcinoids.

## Methods

A retrospective search of a prospectively maintained electronic database of the Modbnury Hospital, a teaching hospital in Australia, was undertaken. International Classification of Diseases (ICD) codes for AP for a 50-month period, from March 2007 to April 2011 were analysed with the aim of identifying all patients admitted to the hospital with acute appendicitis who underwent emergency appendicectomies. This resulted in a total of 506 patients being identified. Each admission was reviewed within the electronic database for patient admission details.

Patients presenting with right lower abdominal pain, consistent examination findings, with supporting blood investigations, such as raised white cell and neutrophil counts and/or the inflammatory marker serum C-reactive protein levels, were considered to have acute appendicitis and proceeded for an appendicectomy (open, laparoscopic and/laparoscopic converted to laparotomy).

In patients in whom the diagnosis was unclear, the use of imaging (ultrasonography or computed tomography scan) was considered prior to planning surgery. Antibiotics (usually a Cephalosporin) were administered at induction.

Laparoscopic appendicectomies were performed using the standard three port technique. In patients in whom the laparoscopic procedure could not be completed safely, a conversion to a laparotomy was performed. All appendicectomies specimens were routinely submitted for histopathological analysis as per the hospital protocol.

In the time trend studies, examining the influence of the number of appendicectomies, the cohort was divided into two groups: Group 1: March 2007 to March 2009; Group 2: April 2009 to April 2011.

Statistical analyses were performed using the Statistical Product and Service Solutions, SPSS 14.0 for Windows. Nominal data is provided as number (%) and continuous data as median (range).

## Results

A total of 506 patients underwent emergency appendicectomies in the study period. Of these, 8 patients (1.6%) were found to have appendiceal carcinoids in addition to acute appendicitis. The clinico-pathological features of these patients have been provided in Table 1. While appendicectomy was the only procedure performed in 7 of the 8 patients, in the only patient with a tumor of 2.5 cm (occurring within 5 mm of the base of the appendix), a right hemicolectomy was performed as an interval procedure.

**Table 1:** Clinico-Pathological Features of the Patient Cohort (n = 8)

Characteristic	
Patient-related factors	
Presenting symptoms	Right iliac fossa pain (100%)
Median duration of symptoms (range in days)	1 (0 - 14 days)
Median age (range in years)	23 (12 - 59 years)
Sex (F:M)	7:1
Tumor-related factors	
Appendicular perforation (%)	2 (25%)
Median Size (range in mm)	5 (2 - 25 mm)
Location of lesion	
Tip	5 (62.5%)
Base	3 (37.5%)
Surgery-related factors	
Type of surgery	
Laparoscopy	6 (75%)
Laparoscopy converted to laparotomy	2 (25%)
Median duration of stay (range in days)	2 (1 - 6 days)
Post-operative morbidity (%)	1 (12.5%)

### Time trends

#### Group 1

A total of 222 patients underwent emergency appendicectomies of which 4 patients (1.8%) were found to have appendiceal carcinoids. The median age of patients in this time cohort was 29.5 years (range 12 - 59 years) with all 4 patients being female. The median tumor size was 5 (2 - 7 mm) with 3 tumors located at the tip and 1 at the base.

#### Group 2

A total of 284 patients underwent emergency appendicectomies of which 4 patients (1.4%) were found to have appendiceal carcinoids. The median age of patients in this time cohort was 21 years (range 14 - 37 years) including 3 female and 1 male patient. The median tumor size was 4 (2 - 25 mm) with 2 tumors located at the tip and 2 at the base. There was no significant difference (P < 0.12) in the incidence of carcinoids between the two groups.

## Discussion

In this study, conducted in a community teaching hospital in South Australia, appendiceal carcinoids were found to occur in 1.6% of emergency appendicectomies performed for acute appendicitis. Most of these were incidental lesions [1] detected only on account of the patients undergoing surgery for acute appendicitis. The high incidence of appendiceal carcinoids in this study (nearly twice the previously reported incidence rate for appendiceal carcinoids [1, 2]) is one of the few reports suggesting an increasing incidence of these lesions [4, 6]. The cause for this increased incidence remains poorly understood. One hypothesis is based on the premise that there is an increase in the number of appendicectomies being performed [4] due to the availability of laparoscopy [7]. However, this study has demonstrated that although the number of appendicectomies performed increased over the two time periods in our hospital, this increase in the number of surgeries did not influence the incidence of appendiceal carcinoids.

The female preponderance amongst the patients with carcinoids noted in this study is similar to previously reported studies [4, 6].

The predilection for benign carcinoids in younger patients (20 - 30 years of age) and their preferential location in the tip of the appendix are well recognised [8] and is attributed to the origin of the tumor from subepithelial neuroendocrine cells [9]. Subepithelial neuroendocrine cells have been found to be more numerous at the tip of the appendix than at the base and their density tended to increase with age: peaking around the third decade followed by another decline with further advancing age [10].

### Conclusions

Appendiceal carcinoids were found in 1.6% of patients undergoing emergency appendicectomy for acute appendicitis. These tumors are found more commonly in young females with a predilection for the tip of the appendix. The perceived increased incidence appendiceal carcinoids appear to be unrelated to the increase in the number of appendicectomies being performed.
